# Whole genome sequencing and phylogenetic analysis of West Nile viruses from animals in New England, United States, 2021

**DOI:** 10.3389/fvets.2023.1085554

**Published:** 2023-04-28

**Authors:** Ji-Yeon Hyeon, Zeinab H. Helal, Allison Appel, Natalie Tocco, Amelia Hunt, Dong-Hun Lee, Guillermo R. Risatti

**Affiliations:** ^1^Department of Pathobiology and Veterinary Science, College of Agriculture, Health and Natural Resources, University of Connecticut, Storrs, CT, United States; ^2^Connecticut Veterinary Medical Diagnostic Laboratory, Department of Pathobiology and Veterinary Science, College of Agriculture, Health and Natural Resources, University of Connecticut, Storrs, CT, United States; ^3^College of Veterinary Medicine, Konkuk University, Seoul, Republic of Korea

**Keywords:** West Nile virus, genome sequencing, phylogenetic analysis, surveillance, epidemiology

## Abstract

West Nile virus is a mosquito-borne Flavivirus which is the leading cause of global arboviral encephalitis. We sequenced WNVs from an American crow found in Connecticut and an alpaca found in Massachusetts which were submitted to the Connecticut Veterinary Medical Diagnostic Laboratory (CVMDL). We report here the complete protein-coding sequences (CDS) of the WNVs (WNV 21-3957/USA CT/Crow/2021 and WNV 21-3782/USA MA/Alpaca/2021) and their phylogenetic relationship with other WNVs recovered from across the United States. In the phylogenetic analysis, the WNVs from this study belonged to the WNV lineage 1. The WNV 21-3957/USA CT/Crow/2021 clustered with WNVs from a mosquito and birds in New York during 2007–2013. Interestingly, the virus detected in the alpaca, WNV 21-3782/USA MA/Alpaca/2021 clustered with WNVs from mosquitos in New York, Texas, and Arizona during 2012–2016. The genetic differences between the viruses detected during the same season in an American crow and an alpaca suggest that vector-host feeding preferences are most likely driving viral transmission. The CDS of the WNVs and their phylogenetic relationships with other WNVs established in this study would be useful as reference data for future investigations on WNVs. Seasonal surveillance of WNV in birds and mammals and the genetic characterization of detected viruses are necessary to monitor patterns of disease presentations and viral evolution within a geographical area.

## 1. Introduction

West Nile virus (WNV) is a neurotropic mosquito-borne Flavivirus genus within the Flaviviridae family (1). Its transmission cycle involves mosquitoes belonging to *Culex* spp. as vectors and birds as amplifying hosts or reservoirs. Some mammalian species including humans and horses are accidental dead-end hosts (1, 2). It was first isolated in Uganda in 1937 and is currently the most widespread arbovirus geographically worldwide due to its spread throughout two continents within 2 years (3, 4).

Partial sequencing of the gene encoding for the envelope protein (E) of WNV led to the classification of the virus into five distinct phylogenetic lineages, and the WNV lineage 1 and WNV lineage 2 have been associated with outbreaks in humans (5, 6). Lineage 1 encompasses viruses from Africa, the Middle East, Eastern Europe, the United States, and Australia. Lineage 2 comprises viruses from sub-Saharan Africa and Madagascar (5).

In the United States, WNV was first detected in New York City in 1999 and spread rapidly across the United States within only a couple of years; New York (1999), Connecticut (2000), Florida (2001), Rocky Mountains and Washington state (2002), and Southern California (2003) (3, 7). It has been suggested that there are multiple possible origins of WNV in the United States, but the most likely explanation is that it was due to the human transportation of birds and/or mosquitoes (7).

Although WNVs are seasonally detected in birds and less frequently in mammals in most of the United States, there is limited phylogenetic data based on whole genome sequences (WGS), impeding a more detailed understanding of WNV evolution. Regionally, like in the Northeastern region of the United States, where WNV was first introduced, there are no reports regarding complete genomes of the virus detected in birds and mammals during the last two decades. WGS-based phylogenetic analysis would be a useful tool to understand the spread and evolution of WNV.

In this study, we report the complete protein-coding sequences (CDS) of WNVs detected at the Connecticut Veterinary Medical Diagnostic Laboratory (CVMDL) in an American crow (*Corvus brachyrhynchos*) from Connecticut and an alpaca (*Vicugna pacos*) from Massachusetts during 2021 using next-generation sequencing (NGS). We analyzed their phylogenetic relationship with other WNVs recovered from across the United States to reconstruct the origin of these viruses.

## 2. Materials and methods

### 2.1. Samples

We found two WNVs from animals submitted to the Connecticut Veterinary Medical Diagnostic Laboratory (CVMDL). An American crow (*Corvus brachyrhynchos*) found dead in Branford, CT, and a female alpaca (*Vicugna pacos*) from the state of Massachusetts were confirmed WNV positive using the quantitative reverse transcription real-time PCR (RT-qPCR) assay (8) at the CVMDL, Department of Pathobiology and Veterinary Science, the University of Connecticut in 2021.

### 2.2. RT-qPCR and whole genome sequencing

Total RNA was extracted from brain tissue samples using the TRIzol reagent (ThermoFisher Scientific, USA) according to the manufacturer's instructions for RT-qPCR. Ct values were 14.31 and 17.18 for the American crow and the alpaca samples, respectively. The RNA samples were then used for whole genome sequencing. Sequence-Independent, Single-Primer-Amplification (SISPA) was performed to amplify viral RNA as described in the previous study (9). The Swift 2S Turbo DNA Library Kits (Swift Biosciences, Coralville, IA) were used according to the manufacturer's instructions to generate multiplexed paired-end sequencing libraries. The dsDNA was fragmented and tagged with adapters by Nextera transposase (Illumina, San Diego, CA). Sequencing libraries were purified using Agencourt AMPure XP beads (Beckman Coulter, Brea, CA) and analyzed on a High Sensitivity DNA Chip on the Bioanalyzer (Agilent Technologies, Santa Clara, CA). The libraries were adjusted to 1 nM concentration and equal volumes of 5 μl of each library were pooled. The pool was denatured with sodium hydroxide (0.2 N final concentration) and further diluted to 100 pM. Five percent of PhiX control library (Illumina) was added to the pool. The library pool was loaded in the flow cell of the MiSeq Reagent Kit V3 (Illumina). The barcoded multiplexed library sequencing (2 × 300 bp) was performed on an Illumina MiSeq platform (Illumina).

### 2.3. Assembly of sequencing reads

Residual adapters, SISPA primer K (GACCATCTAGCGACCTCCAC), and bases with low-quality scores (Q < 20) were removed from fastq files using BBduk. Then, reference-guided genome assemblies against reference genome sequences (GenBank accession number: KX547196 and KX547200) were performed using the Minimap2 in Geneious Prime 10 Software (https://www.geneious.com/) and the consensus genome sequences were called using the Geneious Prime 10 with default parameter settings, hereafter referred to as WNV 21-3782/USA MA/Alpaca/2021 and WNV 21-3957/USA CT/Crow/2021 virus.

### 2.4. Phylogenetic analysis

The CDS of WNVs identified in the United States which have metadata including host and collection date (*n* = 902), WNV lineage 1 reference sequence (NC 009942), and WNV lineage 2 reference sequence (NC 001563) were downloaded from NCBI GenBank database. The ElimDupes software (http://hcv.lanl.gov/content/sequence/ELIMDUPES/elimdupes.html) was used to down-sample the data set of 902 WNVs with 99.5% sequence similarity cutoff level to 140 sequences. Two reference sequences and our sequences were added to the datasets for phylogenetic analysis.

The MAFFT multiple alignment software v1.4.0 in Geneious was used for multiple sequence alignment of complete CDS of the WNV genomes. Maximum likelihood (ML) phylogenies were constructed using RAxML-HPC v.8 using the general time-reversible (GTR) nucleotide substitution model and discrete gamma distribution with 1,000 rapid bootstrap replicates, and TempEst v1.5.3 was used to identify potential outliers that substantially deviated from the linear regression of root-to-tip genetic distance against time, and the outliers were removed from this study (10, 11). Phylogenetic trees were rooted to the WNV lineage 2 reference sequence (NC 001563) as an outgroup. Subtrees including the WNVs from this study were extracted from ML phylogenies to better visualize the genetic relationships.

To investigate amino acid changes, the CDS of WNVs were annotated using the “find annotations” feature in the Geneious prime by comparing with the WNV strain HNY1999 polyprotein gene (Accession no. AF202541) and translated. Amino acid mutations in the molecular markers for virulence determinants in mammalian and avian hosts reported in a previous study (12) were investigated.

## 3. Results

The length of sequenced WNV genomes was 10,533 bp, and nucleotide pairwise identity among the two sequences was 98.4% (data not shown). The NCBI BLAST searches revealed that WNV 21-3782/USA MA/Alpaca/2021 virus shared high nucleotide identity (>99.0%) with WNVs identified from mosquitos, birds, and humans in the United States between 2010 and 2014 (Table 1), whereas WNV 21-3957/USA CT/Crow/2021 virus shared nucleotide identity with WNVs identified from mosquitos and a bird in the United States between 2012 and 2015 (Table 2).

**Table 1:** Nucleotide sequence identities between the WNV 21-3782/USA MA/Alpaca/2021 virus and nearest virus homologs in the GenBank database (as of 15 May 2022).

**Accession no**.	**Virus name**	**Collection date**	**Location**	**Host**	**% identity**
KX547196.1	West Nile virus strain WNV-1/Culex/USA/13290644/2013	17-Sep-2013	USA: Nassau Co., NY	*Culex sp*.	99.52%
KY782106.1	West Nile virus isolate B3	25-Jul-2012	USA: Illinois, West Chicago suburbs	*Spinus tristis*	99.48%
KX547167.1	West Nile virus strain WNV-1/Culiseta sp./USA/13330613/2013	04-Sep-2013	USA: Onondaga Co., NY	*Culiseta sp*.	99.47%
KX547565.1	West Nile virus strain WNV-1/Culiseta sp./USA/13330653/2013	24-Sep-2013	USA: Onondaga Co., NY	*Culiseta sp*.	99.46%
KM012188.1	West Nile virus isolate ARC13-12	2012	USA: IL	*Homo sapiens*	99.38%
KX547391.1	West Nile virus strain WNV-1/Culex/USA/10140626/2010	25-Aug-2010	USA: Erie Co., NY	*Culex sp*.	99.38%
KX547485.1	West Nile virus strain WNV-1/Culex/USA/14430021/2014	13-Jun-2014	USA: Rockland Co., NY	*Culex sp*.	99.37%
KX547556.1	West Nile virus strain WNV-1/Culiseta sp./USA/12370596/2012	06-Sep-2012	USA: Oswego Co., NY	*Culiseta sp*.	99.35%
KX547473.1	West Nile virus strain WNV-1/Culex/USA/13141233/2013	17-Jul-2013	USA: Erie Co., NY	*Cultex sp*.	99.35%
KC736498.1	West Nile virus isolate AVA1204579	2012	USA: Texas	*Culex quinquefasciatus*	99.35%

**Table 2:** Nucleotide sequence identities between the WNV 21-3957/USA CT/Crow/2021 virus and nearest virus homologs in the GenBank database (as of 15 May 2022).

**Accession no**.	**Virus name**	**Collection date**	**Location**	**Host**	**% identity**
KX547235.1	West Nile virus strain WNV-1/Culex sp./USA/15350183/2015	13-Aug-2015	USA: Orange Co., NY	*Culex sp*.	99.60%
KX547482.1	West Nile virus strain WNV-1/Culiseta sp./USA/13370456/2013	07-Aug-2013	USA: Oswego Co., NY	*Culiseta sp*.	99.44%
KX547254.1	West Nile virus strain WNV-1/Culex/USA/13510557/2013	17-Jul-2013	USA: Suffolk Co., NY	*Culex sp*.	99.43%
KC333376.1	West Nile virus isolate TX8546	14-Jun-2012	USA: Katy, Harris Co., Texas	*blue jay*	99.43%
KX547337.1	West Nile virus strain WNV-1/Culex/USA/12430655/2012	28-Sep-2012	USA: Rockland Co., NY	*Culex sp*.	99.42%
KX547333.1	West Nile virus strain WNV-1/Culex/USA/13511447/2013	09-Oct-2013	USA: Suffolk Co., NY	*Culex sp*.	99.42%
KX547201.1	West Nile virus strain WNV-1/Aedes sp./USA/12290391/2012	20-Jul-2012	USA: Nassau Co., NY	*Aedes sp*.	99.42%
KY216153.1	West Nile virus isolate 557	25-Jul-2012	USA: Not further specified	*Culex pipiens*	99.41%
KY229073.1	West Nile virus isolate 875	21-Aug-2012	USA: Not further specified	*Culex pipiens*	99.39%
KX547612.1	West Nile virus strain WNV-1/Culex/USA/12510875/2012	21-Aug-2012	USA: Suffolk Co., NY	*Culex sp*.	99.39%

**Figure 1:**
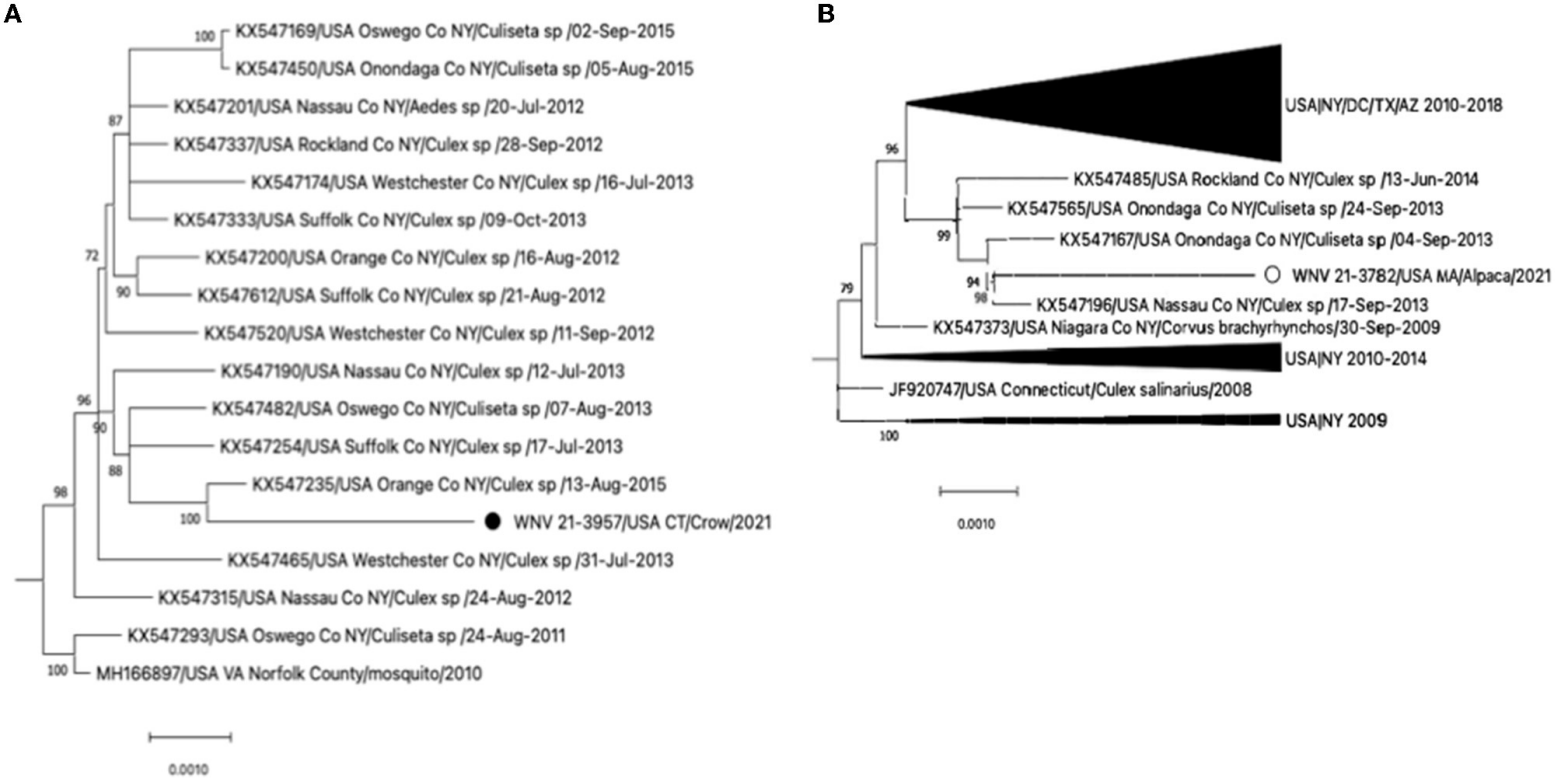
**(A)** Expansion of the clade of WNV 21-3957/USA CT/Crow/2021 and **(B)** the clade of WNV 21-3957/USA CT/Crow/2021 from the full Maximum-likelihood tree in Supplementary Figure 2. The black circle and open circle identify WNV 21-3957/USA CT/Crow/2021 and WNV 21-3957/USA CT/Crow/2021 virus, respectively. The scale bars show the number of substitutions per site.

The phylogenetic analysis revealed that WNV 21-3782/USA MA/Alpaca/2021 and WNV 21-3957/USA CT/Crow/2021 belonged to the WNV lineage 1 (Supplementary Figures 1, 2). The WNV 21-3957/USA CT/Crow/2021 clustered with WNVs detected in mosquitoes in New York during 2013-2015 (Figure 1A). This virus was not genetically related to the WNVs detected in American crows in Connecticut during 1999–2002 (Supplementary Figures 1, 2). The WNV 21-3782/USA MA/Alpaca/2021 clustered with WNVs detected in mosquitos in New York from 2013 to 2014 (Figure 1B).

We investigated if predicted amino acid substitutions observed in the CDS of WNV 21-3957/USA CT/Crow/2021 and WNV 21-3782/USA MA/Alpaca/2021 sequences encompass previously identified molecular markers of virulence in WNV (12). The CDS of WNV 21-3782/USA CT/Alpaca/2021 harbors amino acid substitutions, including E-159A and NS4A-46E/47L/50A, but the amino acid sequences were different from the previous study (Table 3). A T249P substitution in non-structural protein 3 (NSP3) associated with decreased virulence in the avian model of WNV virulence was detected in WNV 21-3957/USA CT/Crow/2021 (Table 4).

**Table 3:** Amino acid substitutions of WNV 21-3782/USA CT/Alpaca/2021 virus in virulence determinants in the WNV genome found in mammalian host.

**Virulence determinants (protein-amino acid substitution)**		**WNV 21-3782/USA CT/Alpaca/2021**
Mutations involved in the attenuated viral phenotype	PrM-N15Q	N
	M-I36F	I
	M-A43G	A
	E-L107F	L
	E-A316V/E	A
	E-K440R	K
	NS1-N130A	N
	NS1-N175A	N
	NS1-N207A	N
	NS1-P250L	P
	NS2A-A30P	A
	NS3-249	P
	NS3-483	D
	NS4A-Q46K	E
	NS4A-Q47K	L
	NS4A-D50K	A
	NS4B-P38G	P
	NS4B-C102S	C
	NS4B-E249G	E
	NS5-K61A	K
	NS5-D146A	D
	NS5-K182A	K
	NS5-E218A	E
	NS5-A804V	A
Mutations involved in the more virulent viral phenotype	E-154	N
	E-156	S
	E-I159V	A

**Table 4:** Amino acid substitutions of WNV 21-3957/USA CT/Crow/2021 virus in virulence determinants in the WNV genome found in avian host.

**Virulence determinants (protein-amino acid substitution)**		**WNV 21-3957/USA CT/Crow/2021**
Mutations involved in the attenuated viral phenotype	PrM-I141T	I
	E-S156P	S
	NS3-483	D
Mutations involved in the more virulent viral phenotype	NS1-K110N	K
	NS3-T249P	P
	NS4A-F92L	F

## 4. Discussion

Since WNV was first detected in the United States in 1999 in New York City, WNV has spread from the initial point of entry in the northeastern United States to the rest of the United States, Mexico, Canada, and the Caribbean (13, 14). Most complete genome sequences of WNVs available in the NCBI GenBank are WNVs from mosquitoes in the northeastern United States and only a few phylogenetic studies have been performed based on full-length genome sequences (13, 15, 16). In this study, we detected WNVs via RT-qPCR in a mammalian host (alpaca) and an avian host (American crow). These animals originated from the New England region (Connecticut and Massachusetts) and were submitted in 2021 to the Pathology section of CVMDL for post-mortem diagnostics. Detected viruses were then genetically characterized via next generation sequencing considering that only a few phylogenetic studies have been based on the WGS of WNV (13, 15, 16). Here, NGS in combination with sequence enrichment steps via SISPA allowed the obtention of complete CDS of WNV directly from clinical samples.

In the ML phylogenetic trees, the long tree branch length between the WNVs sequenced in this study and their closest relatives suggest that the virus had been circulating before being detected in the crow and alpaca in 2021. We assume that the WNVs have been maintained in mosquitoes and were transmitted to these animals. In addition, the root-to-tip regression analysis of ML phylogeny using 904 complete genome sequences of WNVs showed a positive correlation between time and genetic distance with a high correlation coefficient value (0.93) and *R* square value (0.87), indicating gradual genetic evolution of WNVs in North America at an estimated evolutionary rate of 4.59 × 10^−4^ substitutions/site/year (data not shown). However, the details of the transmission routes of these viruses remain uncertain due to the lack of recent genome sequences and surveillance data.

Both the American crow and the alpaca had a history of neurological signs preceding death. Considering the seasonality of the disease, WNV was considered one of the diagnostics differentials for both animals. WNV is considered a major public and animal health problem, causing diverse pathologies ranging from mild febrile to severe neurological damage and death. WNV pathotypes in birds and mammals have been associated with specific genotypes (12). For instance, the NS3-T249P mutation observed in WNV 21-3957/USA CT/Crow/2021 is a critical determinant of WNV virulence in American crows and present in many WNV strains that caused major outbreaks in humans such as in Egypt (1950), Romania (1996), Russia (1996), New York (1999), and Israel (1997–1998) (12, 17, 18). In Brault et al.'s studies (17, 18), the mutant WNV NY99-P249T and KN3829 (attenuated strain) gave rise to a low level, delayed viremia at day 3 pi, compared to high titers observed in WNV NY99 and KN3829-T249P. In addition, the E-159A observed in the WNVs sequenced in this study was found in many WNVs recovered after 2001, suggesting a possible link with the enhanced WNV spread and pathology in America after the year 2000 (19). Mutations at the NS4A sites have been associated with increased WNV virulence in mammals (12). The identification of virulence determinants and mutants as determined here via WGS is a crucial step in understanding WNV epidemiology, transmission, and pathogenesis. The study conducted here highlights the need for enhanced genomic surveillance of WNVs.

In this study, we report the complete CDS of WNVs identified from a crow and an alpaca in New England in 2021. NGS in combination with SISPA approach enabled WGS of WNVs directly from clinical samples. The use of the described NGS approach will allow efficient complete genome sequencing of circulating WNVs that can provide abundant information to understand the evolution and spread of WNVs. Additionally, the complete genome sequences and their phylogenetic relationships with other WNVs established in this study would be useful as reference data for future genomic surveillance of WNVs. Continued surveillance and genome sequencing of WNVs from animals as well as mosquitos would be needed to monitor virus evolution and transmission and to assess the emergence of genetic mutations that may be relevant for public health.

## Data availability statement

The data presented in the study are deposited in GenBank under the accession number ON994909 and ON994910.

## Ethics statement

Ethical review and approval was not required for the study on animals in accordance with the local legislation and institutional requirements.

## Author contributions

J-YH: WGS data analysis and manuscript writing. ZH, AA, NT, and AH: sample preparation and data collection. D-HL: study design, WGS data analysis, and manuscript editing. GR: supervision and data collection. All authors contributed to the article and approved the submitted version.

## Supplementary material

Supplementary Figure 1 Maximum-likelihood analysis of WNV lineage 1 and 2 reference sequences and 140 complete genome sequences of WNVs identified in United States including two WNVs of this study. The scale bars show the number of substitutions per site. The numerical values represent 1,000 bootstrap replicate values expressed as a percentage. The WNVs sequenced in this study were highlighted in red. Phylogenetic tree was rooted to the WNV lineage 2 reference sequence (NC 001563) as an outgroup.

Supplementary Figure 2 Maximum-likelihood analysis of WNV lineage 1 and 2 reference sequences and 906 complete genome sequences of WNVs identified in United States including two WNVs of this study. The scale bars show the number of substitutions per site. The numerical values represent 1,000 bootstrap replicate values expressed as a percentage. The WNVs sequenced in this study were highlighted in red. Phylogenetic tree was rooted to the WNV lineage 2 reference sequence (NC 001563) as an outgroup.
