# Supplementary figures and images for: Whole genome sequencing and phylogenetic analysis of West Nile viruses from animals in New England, United States, 2021
Source: Front Vet Sci. 2023 Apr 28;10:1085554. doi: 10.3389/fvets.2023.1085554 (PMC10175668; doi:10.3389/fvets.2023.1085554)

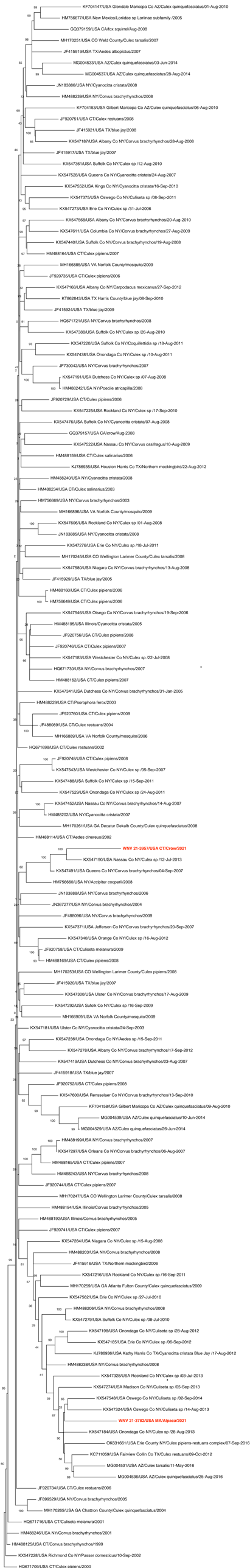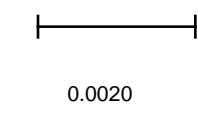

Supplement: Supplementary Figure 1 — Maximum-likelihood analysis of WNV lineage 1 and 2 reference sequences and 140 complete genome sequences of WNVs identified in United States including two WNVs of this study. The scale bars show the number of substitutions per site. The numerical values represent 1,000 bootstrap replicate values expressed as a percentage. The WNVs sequenced in this study were highlighted in red. Phylogenetic tree was rooted to the WNV lineage 2 reference sequence (NC 001563) as an outgroup. [file Data_Sheet_1.PDF]
